# P-2028. Therapeutic Effect of Delayed Treatment with a Second-Generation 3CL Protease Inhibitor S-892216 in Hamsters Infected with SARS-CoV-2

**DOI:** 10.1093/ofid/ofae631.2184

**Published:** 2025-01-29

**Authors:** Masaaki Nakashima, Haruaki Nobori, Alice Shimba, Satoshi Miyagawa, Akane Hayashi, Kazumi Matsumoto, Kaoru Baba, Keita Fukao

**Affiliations:** Shionogi&Co., Ltd., Toyonaka, Osaka, Japan; SHIONOGI & CO., LTD., Toyonaka-shi, Osaka, Japan; Shionogi&Co., Ltd., Toyonaka, Osaka, Japan; Shionogi&Co., Ltd., Toyonaka, Osaka, Japan; Shionogi TechnoAdvance Research, Co., Ltd., Toyonaka, Osaka, Japan; Shionogi TechnoAdvance Research, Co., Ltd., Toyonaka, Osaka, Japan; Shionogi TechnoAdvance Research, Co., Ltd., Toyonaka, Osaka, Japan; Shionogi&Co., Ltd., Toyonaka, Osaka, Japan

## Abstract

**Background:**

SARS-CoV-2 and COVID-19 remain a major global health challenge. We have discovered a second-generation small molecule 3CL protease inhibitor S-892216 that is proceeding with phase 1 study in Japan. S-892216 has potent antiviral activity and addresses current treatment concerns of currently available antivirals regarding drug-drug interaction (DDI) and safety. We evaluated the therapeutic effect of S-892216 by using SARS-CoV-2 infected hamster model. In this study, virus titer in lungs and nasal turbinates (NT), lung inflammation, body weight loss and lung weight increase caused by SARS-CoV-2 infection were analyzed.

**Methods:**

Syrian hamsters were intranasally inoculated with SARS-CoV-2 Omicron BE.1/BA.5-like. The hamsters were orally treated twice daily with S-892216 0.1, 1, and 10 mg/kg or ensitrelvir 50 mg/kg, or subcutaneously treated with nirmatrelvir 750/250 mg/kg (750 mg/kg for the loading dose and 250 mg/kg for the maintenance doses) from 1 day post infection for 5 days. Lungs and NT were collected for virus titers and lung inflammation evaluation. Lung weights were measured 7 days post infection and body weight changes were monitored until 14 days post infection.

**Results:**

Oral treatment with S-892216 significantly reduced virus titers in lung (1.9- and 2.9-log reduction at the doses of 1 and 10 mg/kg, respectively) and NT (1.8- and 2.3-log reduction at the doses of 1 and 10 mg/kg, respectively) 3 days post infection. In addition, S-892216 suppressed lung inflammation and body weight loss caused by SARS-CoV-2 infection dose dependently. The virus titer reduction in lung and NT and suppression of lung inflammation and body weight losses were comparable in S-892216 1 mg/kg, ensitrelvir 50 mg/kg and nirmatrelvir 750/250 mg/kg treatment groups.

**Conclusion:**

Oral treatment with S-892216 exhibited therapeutic effect in SARS-CoV-2 infected hamsters. Furthermore, S-892216 showed comparable efficacy at lower dosages compared to ensitrelvir and nirmatrelvir. These findings suggest that S-892216 could be used at lower dosage than the current treatment options in clinical settings. We will evaluate DDI and the safety potential of S-892216 in clinical trials in future.

**Disclosures:**

Masaaki Nakashima, PhD, Shionogi&Co., Ltd.: Employee|Shionogi&Co., Ltd.: Stocks/Bonds (Private Company) Haruaki Nobori, PhD, Shionogi & Co., Ltd.: Employee|Shionogi & Co., Ltd.: Stocks/Bonds (Private Company) Alice Shimba, n/a, Shionogi & Co., Ltd.: Employee|Shionogi & Co., Ltd.: Stocks/Bonds (Private Company) Satoshi Miyagawa, n/a, Shionogi & Co., Ltd.: Employee|Shionogi & Co., Ltd.: Stocks/Bonds (Private Company) Akane Hayashi, n/a, Shionogi & Co., Ltd.: Stocks/Bonds (Private Company)|Shionogi TechnoAdvance Research: Employee Kazumi Matsumoto, n/a, Shionogi TechnoAdvance Research: Employee Kaoru Baba, n/a, Shionogi TechnoAdvance Research: Employee Keita Fukao, MD, SHIONOGI and CO., LTD.: Stocks/Bonds (Private Company)

